# Supplementary material for: Circulating Lymphocytes Reflect the Local Immune Response in Patients with Colorectal Carcinoma
Source: Diagnostics (Basel). 2022 Jun 7;12(6):1408. doi: 10.3390/diagnostics12061408 (PMC9221878; doi:10.3390/diagnostics12061408)
Supplement: Supplementary file 1 [file diagnostics-12-01408-s001.zip › diagnostics-1756040-supplementary.pdf]

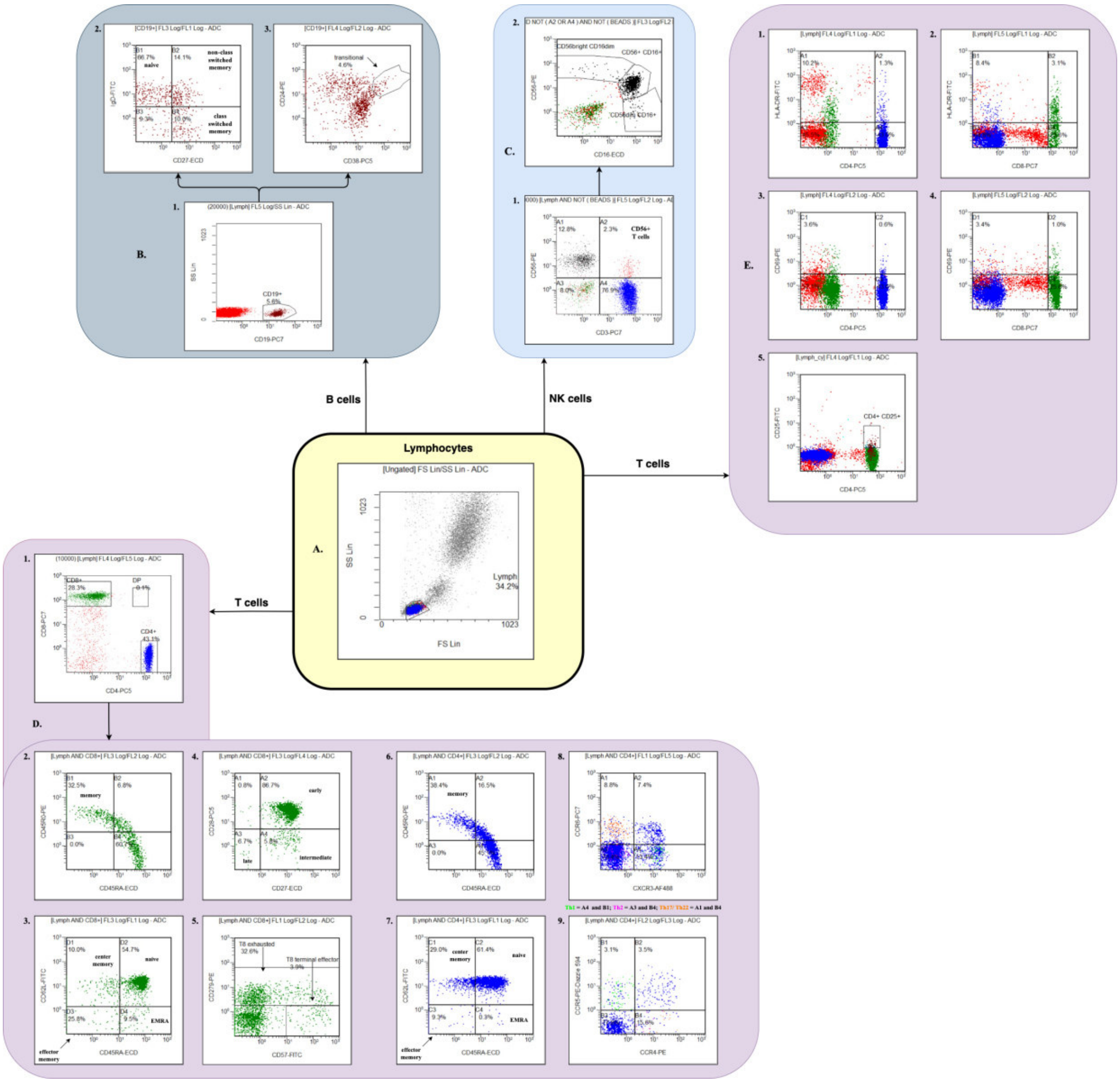

Figure S1. Gating Strategy. (**A**.) Lymphocytes were identified by using forward scatter (FC) and side scatter (SC). (**B**.) Additional CD19 positivity was used to define B cells (Plot 1), which were further subdivided into naïve (IgD+), class switched memory (CD27+), non-class switched memory (IgD+ CD27+, Plot 2) and transitional B cells (CD24+ CD38+, Plot 3). (**C**.) NKT cells (CD3+ CD56+) were primarily identified (Plot 1). CD3- cells were classified into 3 different subgroups of NK cells (CD56+ CD16+, CD56bright CD56 dim, CD56dim CD16bright, Plot 2).

(D.) T cells were identified by CD4 or CD8 positivity. Both CD4+ as well as CD8+ cells were subdivided into memory (CD45RA- CD45RO+) (Plots 1+6), naïve (CD62L+ CD45RA+), central memory (CD62L+ CD45RA-), effector memory (CD62L- CD45RA-) and effector memory RA+ (“EMRA”) (CD62L- CD45RA+) cells (Plots 3+7). To analyze CD8+ cell activity, cells were subdivided into early (CD27+ CD28+), intermediate (CD27+ CD28-), late (CD27- CD28-) (Plot 4), or exhausted (CD279+) and terminal effector (CD279- CD57+) cells (Plot 5). CD4+ T helper cells were classified into Th1 (CXCR3+ CCR4- CCR5+ CCR6-), Th2 (CXCR3- CCR4+ CCR5- CCR6-) and Th17/Th22 (CXCR3- CCR4+ CCR5- CCR6+) cells (Plots 8+9). **(E.)** CD4+ and CD8+ cells were also subdivided into activated cells (HLA-DR+ or CD69+) (Plots 1-4) and regulatory cells (CD4+ CD25high) (Plot 5).

Table S1: Fluorochrome-antibody conjugates

Cell population	Antigen	Fluorochrome	Clone	Isotype	Beckman Coulter/Biolegend item number
B	IgD	FITC	IA6-2	IgG2a	B30652
B	CD24	PE	ALB9	IgG1	IM1428U
B, exhausted T	CD27	ECD	1A4CD27	IgG1	B26603
B	CD38	PC5	LS198-4-3	IgG1	A07780
B	CD19	PC7	J3-119	IgG1 kappa	IM3628
Memory T	CD62L	FITC	DREG56	IgG1	IM1231U
Memory T	CD45 R0	PE	UCHL1	IgG2a	A07787
Memory T, Treg	CD45RA	ECD	2H4LDH11LDB9	IgG1	IM2711U
Memory T, activated T, Treg, Th subsets	CD4	PC5	13B8.2	IgG1	A07752
Memory T, Treg, exhausted T, activated T	CD8	PC7	SFCI21Thy2D3	IgG1	737661
Exhausted CD8-T	CD57	FITC	NC1	IgM	B49188
Exhausted CD8-T	CD279	PE	PD1.3	IgG2b	B30634
Exhausted CD8-T	CD28	PC5	CD28.2	IgG1	6607108
Activated T	HLA-DR	FITC	Immu-357	IgG1	IM1638U
Activated T	CD69	PE	TP1.55.3	IgG2b	IM1943U
Treg	CD25	FITC	B1.49.9	IgG2a	IM0478U
Treg	FoxP3	PE	Ab259D	IgG1 kappa	B46031
T/NK	CD56	PE	N901/NK-1	IgG1	A07788
T/NK	CD16	ECD	3G8	IgG1	A33098
T/NK	CD19	PC5	J3-119	IgG1	A07771
T/NK	CD3	PC7	UCHT1	IgG1	737657
Th subsets	CD183 (CXCR3)	AF488	G025H7	IgG1	B68144
	CD194 (CCR4)	PE	L291H4	IgG1	359412
	CD195 (CCR5)	PE/Dazzle 594	J418F1	IgG2b	359126
	CD196 (CCR6)	PC7	B-R35	IgG2a	B68132

FITC fluorescein isothiocyanate, *PE* phycoerythrin, *ECD* phycoerythrin-Texas red, *PC-5* phycoerythrin cyanin, *PC7* phycoerythrin cyanin7, *AF488* Alexa-Fluor 488
